# Back-Up Base Excision DNA Repair in Human Cells Deficient in the Major AP Endonuclease, APE1

**DOI:** 10.3390/ijms25010064

**Published:** 2023-12-20

**Authors:** Daria V. Kim, Evgeniia A. Diatlova, Timofey D. Zharkov, Vasily S. Melentyev, Anna V. Yudkina, Anton V. Endutkin, Dmitry O. Zharkov

**Affiliations:** 1 Siberian Branch of the Russian Academy of Sciences Institute of Chemical Biology and Fundamental Medicine, 8 Lavrentieva Ave., 630090 Novosibirsk, Russia; dkim@niboch.nsc.ru (D.V.K.); diatlova@niboch.nsc.ru (E.A.D.); timazharkov74@gmail.com (T.D.Z.); vmelentiyev@gmail.com (V.S.M.); ayudkina@niboch.nsc.ru (A.V.Y.); aend@niboch.nsc.ru (A.V.E.); 2 Department of Natural Sciences, Novosibirsk State University, 2 Pirogova St., 630090 Novosibirsk, Russia

**Keywords:** DNA repair, abasic sites, uracil, AP endonucleases, DNA glycosylases, APE1, APE2, NTHL1

## Abstract

Apurinic/apyrimidinic (AP) sites are abundant DNA lesions generated both by spontaneous base loss and as intermediates of base excision DNA repair. In human cells, they are normally repaired by an essential AP endonuclease, APE1, encoded by the *APEX1* gene. Other enzymes can cleave AP sites by either hydrolysis or β-elimination in vitro, but it is not clear whether they provide the second line of defense in living cells. Here, we studied AP site repairs in *APEX1* knockout derivatives of HEK293FT cells using a reporter system based on transcriptional mutagenesis in the enhanced green fluorescent protein gene. Despite an apparent lack of AP site-processing activity in vitro, the cells efficiently repaired the tetrahydrofuran AP site analog resistant to β-elimination. This ability persisted even when the second AP endonuclease homolog, APE2, was also knocked out. Moreover, *APEX1* null cells were able to repair uracil, a DNA lesion that is removed via the formation of an AP site. If AP site hydrolysis was chemically blocked, the uracil repair required the presence of NTHL1, an enzyme that catalyzes β-elimination. Our results suggest that human cells possess at least two back-up AP site repair pathways, one of which is NTHL1-dependent.

## 1. Introduction

Spontaneous base loss from DNA mostly affects purine deoxynucleotides and yields apurinic/apyrimidinic (AP) sites [1]. This reaction occurs at non-negligible rates under physiological conditions, producing an estimated background level of ~5–20/10^6^ lesions per human cell per day [2]. AP sites are highly cytotoxic and mutagenic due to their non-instructive nature and chemical instability [3,4]. Thus, fast repair of AP sites is critical in all living organisms to ensure their genome integrity.

AP sites are predominantly removed through the base excision DNA repair (BER) pathway [5,6,7]. As its name implies, this pathway starts with the excision of damaged bases by a collection of DNA glycosylase enzymes. AP sites formed as products of this reaction, as well as the spontaneously arising AP sites, are recognized by AP endonucleases, which hydrolyze their 5′-phosphodiester bonds. The following events may proceed along different subpathways: short-patch repair, long-patch repair with or without DNA polymerase switch, and 5′-gap repair, reviewed in [5,6,7,8], which all ultimately come down to repair DNA synthesis and nick ligation to restore the covalently continuous DNA.

Human cells possess a single major AP endonuclease, APE1 (also known as APEX1, HAP1, or Ref-1), encoded by the *APEX1* gene [9,10]. It is the first common player in BER, starting with different nucleobase or abasic lesions and is extensively connected with many other cellular pathways, ultimately linking BER to transcription activation [11,12,13,14,15,16], active demethylation [17,18], chromatin remodeling [19,20], telomere maintenance [21,22], RNA quality control [23,24], mRNA turnover [25], microRNA processing [26], and programmed cell death [27]. The germline knockout of *Apex1* in mice and the morpholino knockdown of the zebrafish and *Xenopus* homologs are embryonic lethal [28,29,30,31,32], while the full-body knockout in adult floxed mice renders them hypersensitive to cerebral ischemia [33]. Brain-specific knockout mice develop normally in utero but die soon after birth from massive oxidative brain damage [34]. Several knockout cell lines of human and mouse origin have been reported, their phenotypes ranging from rather mild sensitivity to genotoxic agents to quick, spontaneous apoptosis [35,36,37,38,39].

While APE1-initiated BER is regarded as the major enzymatic pathway for AP site repair in human cells, several possible backup pathways have been considered. DNA glycosylases with AP lyase activity (NTHL1, OGG1, NEIL1, NEIL2, and NEIL3 in human cells) are obviously the first candidates since their ability to cleave natural AP sites is well-documented [40,41]. NTHL1 and OGG1 convert AP sites to 3′-terminal phospho-α,β-unsaturated aldehydes (PUA) that are substrates for APE1 but can also be removed by tyrosyl–DNA phosphodiesterase 1 (TDP1) [42] and possibly other 3′-phosphodiesterases/exonucleases such as APE2 [43]. TDP1 also can cleave internally located AP sites, albeit preferring those in single-stranded DNA [44]. NEIL proteins produce a mixture of the unsaturated aldehyde and 3′-terminal phosphate, which is processed by polynucleotide kinase/3′-phosphatase (PNKP) afterward, with no involvement of APE1 [45]. APE2 (a homolog of APE1), aprataxin- and PNKP-like factor (APLF), and human homologs of *E. coli* TatD 3′→5′ ssDNA/RNA exonuclease (TATDN1 and TATDN3) all possess weak AP site cleavage activity in vitro, but their main function appears to be in the end processing at DNA breaks [43,46,47]. Finally, the removal of AP site analogs by human NER, albeit at a low level, was reported [48], and genetic evidence in yeast points to NER as a backup system for AP site repair [49]. In particular, RNA polymerase stalling at AP sites was shown to trigger TC-NER in yeast [50], whereas in human cells, the complementary GG-NER pathway is more efficient than TC-NER at this [51]. Indeed, the UV-DDB damage sensor protein binds AP sites with nanomolar affinity and can stimulate either NER or BER [52,53,54]. Nevertheless, the relationships between the different proposed mechanisms during the AP site repair in cells remain poorly understood.

Plasmid reporter constructs with specifically placed lesions offer an unprecedented way to study mutagenesis and DNA repair in situ [55,56]. One recently developed approach relies on using restriction endonucleases with a nicking activity that have recognition sites located in tandem with the reporter gene. In particular, the *eGFP* coding sequence contains two recognition sites for Nb.Bpu10I and Nt.Bpu10I nickases, allowing the introduction of lesions into both the transcribed and coding strands [57]. This reporter system, combined with cells deficient in certain repair genes, was used to show that thymine glycol lesions are removed by NTHL1-initiated BER and that NER contributes to the repair of AP sites [51]. Similar systems based on fluorescent protein reporters were employed to analyze the activity of cancer-associated variants of MUTYH DNA glycosylase [58], to address the role of TCR in the repair of etheno adducts [59], to survey the population variability of BER enzymes at a single-cell level [60], etc. Here, we have applied such an EGFP system to obtain evidence for back-up repair pathways of AP sites, one of which depends on NTHL1 DNA glycosylase/AP lyase for AP site processing.

## 2. Results

### 2.1. Plasmid Reporter Systems to Study Base Excision Repair in Cellulo

**Figure 1:**
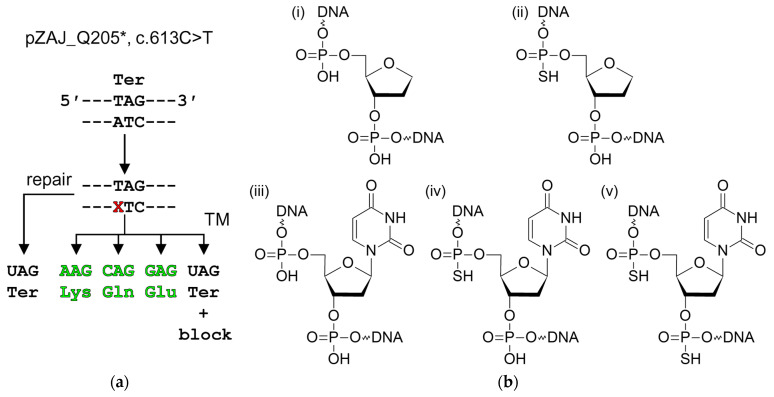
(**a**) Scheme of site-specific lesion (X) positioning and the repair and transcription mutagenesis (TM) outcomes in the EGFP reporter construct pZAJ_Q205* [51]. Green codons and amino acid residue names indicate fluorescent EGFP variants. Ter, stop codon. (**b**) Structures of DNA lesions studied in this work: (i) F; (ii) sF; (iii) U; (iv) sU; (v) sUs.

Systems based on plasmids with lesions introduced into a reporter gene are valuable tools for studying repair in living cells. In one particularly useful design, a lesion is placed in a dysfunctional reporter and detects the level of transcriptional mutagenesis (TM, misincorporation of NMPs opposite the lesion by an RNA polymerase) that restores the activity [51,61]. For example, a c.613C > T mutation in the *eGFP* gene replaces the 5′-CAG-3′ Gln205 codon with the 5′-TAG-3′ stop codon, leading to a loss of fluorescence, while any amino acid potentially arising from the TAG codon by a single-nucleotide substitution (Gln, Glu, Leu, Lys, Ser, Trp, or Tyr) produces fluorescent protein (Figure 1a). If a lesion is placed in the template strand opposite to T, some transcripts will encode a fluorescent EGFP by misreading, while other readthrough events together with transcription blockage events will produce a non-fluorescent protein. In any case, faithful DNA repair will restore the original sequence of the truncated non-fluorescent variant. Thus, the efficiency of repair is inversely proportional to the observed fluorescence.

To study the repair potential in human cells, we have used constructs carrying an AP site analog (3-hydroxytetrahydrofuran-2-yl)methyl phosphate (F), F with a 5′-phosphorothioate bond (sF), U, and U with a 5′-phosphorothioate bond (sU) or both 5′- and 3′-phosphorothioate bonds (sUs) (Figure 1b). The presence of the 5′-phosphorothioate bond strongly blocks the hydrolysis by AP endonuclease [62,63], while the 3′-phosphorothioate bond suppresses—but does not fully abolish—the action of AP lyases from different structural families [64,65,66]. A combination of these modified nucleotides allows the dissection of the chemical nature of the reactions taking place during the repair.

### 2.2. AP Sites Are Repaired in APEX1 Knockout Cells

**Figure 2:**
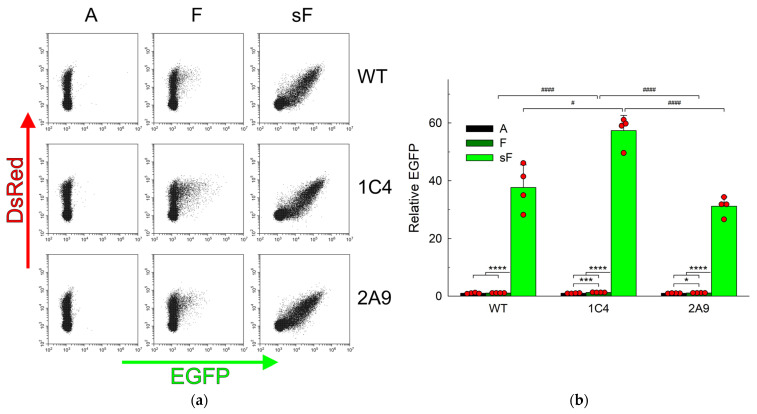
Abasic site-induced transcriptional mutagenesis in wild-type HEK293FT cells and its *APEX1^KO^* derivatives 1C4 and 2A9. (**a**) Representative FACS plots of fluorescence in the green and red channels in cells co-transfected with undamaged pDsRed-Monomer-N1 and pZAJ_Q205* carrying an undamaged A or a lesion. (**b**) Relative EGFP expression normalized for the fluorescence of the control A-construct (*n* = 4, mean ± SD shown). Differences between constructs: *p* < 0.05 (*); *p* < 0.005 (***); *p* < 0.001 (****). Differences between genotypes: *p* < 0.05 (#); *p* < 0.001 (####).

Since APE1 is the main AP endonuclease in human cells [67,68], the repair of AP sites was first studied in wild-type HEK293FT human cell line and its *APEX1* knockout (*APEX1^KO^*) descendants, 1C4 and 2A9 [39]. These cells are devoid of the protein detectable by Western blots and of the activity-cleaving double-stranded oligonucleotides carrying a natural AP site or an F analog but are viable and cycle normally [39]. Given that the natural aldehydic AP sites are unstable, we used constructs based on the pZAJ_Q205* plasmid containing a synthetic analog of the AP site, namely F or sF, resistant to spontaneous β-elimination. Incorporation of any ribonucleotide but U opposite the non-instructive F or sF is expected to produce the fluorescent EGFP, while the repair restores the premature stop codon and results in the truncated non-fluorescent protein (Figure 1a). We first confirmed that under the conditions when wild-type cell extracts convert most of the covalently closed plasmid into the nicked form, the extracts of 1C4 and 2A9 cells show essentially no cleavage of the F-containing plasmid over the background (Supplementary Figure S1). A construct with undamaged A (encoding a non-fluorescent EGFP) prepared in the same way as lesion-containing plasmids was used as a control, and the results were normalized for its fluorescence. Based on the fluorescence distribution in the cells transfected with F-constructs, one can observe the accumulation of a minor population of fluorescent cells in the green channel in wild-type cells (Figure 2a). However, this difference did not reach statistical significance (*p* = 0.33), indicating the efficient repair of this abasic site analog (Figure 2b).

In the pool of *APEX1^KO^* cells transfected with F-constructs, the population of fluorescent cells increased slightly, but cells with the background level of EGFP fluorescence still prevailed (Figure 2a), suggesting that the abasic lesion in the transcribed strand was efficiently repaired and replaced with A, even in the absence of APE1, restoring the non-fluorescent truncated protein. Quantification of these data revealed that the TM level for F-constructs was significantly higher than for control A-constructs in both 1C4 and 2A9, but the magnitude of the effect was low (30% and 12% increase, respectively). Compared with the wild-type cells, the increase in the F-construct fluorescence was significant only in 1C4 cells but not in 2A9 or the pooled 1C4 and 2A9 data (Figure 2b). These results point to the existence of a backup repair pathway, at least for the F abasic sites, in HEK293FT cells.

With the sF-construct, the situation was strikingly different. Even in the WT cells, strong green fluorescence was observed (a 35-fold increase over F), indicating that, first, sF is resistant to repair and, second, that RNA polymerase II efficiently transcribes through it with misincorporation of A, G, or C, producing fluorescent EGFP, either wild-type or the Q205K or Q205E variants (Figure 2b). This observation is consistent with an earlier report in MRC-5 human fetal fibroblasts where sF moderately suppressed transcription and resulted in mutagenic RNA polymerase II bypass [51] and with in vitro data on the bypass of F by yeast and mammalian RNA polymerase II [69]. In both knockout lines, the repair was also significantly suppressed (44-fold fluorescence increase over F in 1C4, 28-fold in 2A9) (Figure 2b). Thus, any F repair backup pathway must be sensitive to the presence of the 5′-phosphorothioate bond, arguing against a possible involvement of NER or other processes that rely on cleaving DNA at a distance from the lesion.

### 2.3. The Backup Repair in APEX1 Knockout Cells Is Independent of APE2

**Figure 3:**
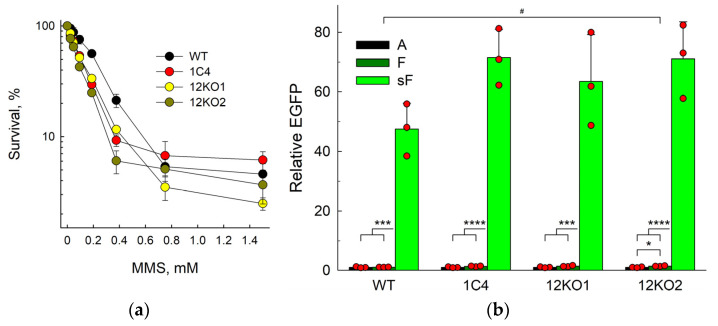
Abasic site-induced transcriptional mutagenesis in wild-type HEK293FT cells and its *APEX1^KO^* and *APEX1^KO^ APEX2^KO^* derivatives. (**a**) Sensitivity of wild-type, 1C4, 12KO1 and 12KO2 lines to MMS. (**b**) Relative EGFP expression normalized for the fluorescence of the control A-construct (*n* = 3, mean ± SD shown). Differences between constructs: *p* < 0.05 (*); *p* < 0.005 (***); *p* < 0.001 (****). Differences between genotypes: *p* < 0.05 (#).

Since the F abasic site analog is resistant to β-elimination and thus cannot be processed by AP lyases, we next addressed the possibility that APE2 can support the residual repair of F in *APEX1* knockout cells. APE2 has recently emerged as an important player in processing blocked 3′-termini critical for survival or BRCA1- and BRCA2-deficient cells [70,71], but its role in AP site repair in vivo remains obscure. From the 1C4 *APEX1^KO^* line, we generated double knockouts, *APEX1^KO^ APEX2^KO^*, using Cas9 editing (Supplementary Figure S2). Two monoclonal lines, 12KO1 and 12KO2, were obtained. Sequencing of thirteen subclones from each line revealed that eight subclones from the 12KO1 cells carried an insertion of a single C (c.49dupC), and five subclones had a 45-bp insertion with an in-frame stop codon (c.50_51insTGCCAGTAACTGTCAGACCCAAGTTCCATGATTTACTTCCCTCCA). All 12KO2 subclones carried an insertion of a single C (c.49dupC). The parental HEK293FT cell line is hypotriploid, so the most likely editing outcome in 12KO1 cells is two c.49dupC alleles and one c.50_51insTGCCAGTAACTGTCAGACCCAAGTTCCATGATTTACTTCCCTCCA allele, while 12KO2 cells contain three c.49dupC alleles. No wild-type allele copies were found among the sequenced subclones, making the probability of missing a wild-type allele in monoclones of hypotriploid HEK293FT cells ~6 × 10^−7^. In both 12KO1 and 12KO2, *APEX2* mRNA was significantly lower than in wild-type (7-and 44-fold, respectively) or 1C4 cells, most likely due to nonsense-mediated mRNA decay often observed in CRISPR/Cas9 gene knockouts. Both lines showed similar sensitivity to methyl methane sulfonate (MMS), a methylating agent that produces AP sites after repair or spontaneous decay of the primary ring-alkylated purine bases, which was not significantly different from the single *APEX1* knockout (Figure 3a).

When transfected with the control A-construct, F-construct, and sF-construct, both *APEX1^KO^ APEX2^KO^* lines demonstrated results very similar to the parent 1C4 *APEX1^KO^* line (Figure 3b). The repair of F was slightly below the wild-type cells (significant only for the 12KO2 clone), whereas the fluorescence in the sF-transfected cells was ~50-fold higher than in the cells transfected with the F-construct. No differences between *APEX1^KO^* and *APEX1^KO^ APEX2^KO^* genotypes were found for any construct. Therefore, it is unlikely that APE2 significantly contributes to the F repair in APE1-null cells.

### 2.4. Uracil in DNA Is Repaired in APEX1 Knockout Cells

**Figure 4:**
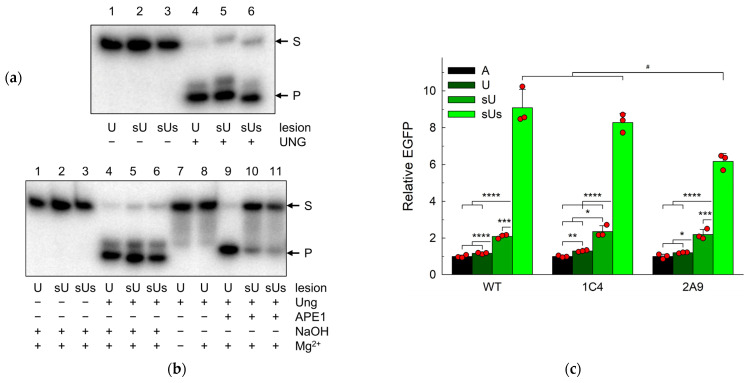
Uracil-induced transcriptional mutagenesis in wild-type HEK293FT cells and its *APEX1* knockout derivatives 1C4 and 2A9. (**a**) Cleavage of U, sU, and sUs by human UNG. (**b**) Cleavage of AP sites generated from U, sU, and sUs by APE1. Lanes 7 and 8 show that Mg^2+^ present in the reaction with APE1 does not substantially contribute to the AP site cleavage. (**c**) Relative EGFP expression normalized for the fluorescence of the control A-construct (*n* = 3, mean ± SD shown). Differences between constructs: *p* < 0.05 (*); *p* < 0.01 (**); *p* < 0.005 (***); *p* < 0.001 (****). Differences between genotypes: *p* < 0.05 (#).

In the classical BER pathway, a DNA glycosylase excises a damaged base and forms a natural AP site, which is later hydrolyzed by an AP endonuclease. Uracil–DNA glycosylases, such as UNG and SMUG1 in human cells, are monofunctional glycosylases yielding the natural AP site in a manner not complicated by further reactions such as β-elimination [72], and APE1 catalyzes the next repair step, the hydrolysis of the phosphodiester backbone 5′ of the AP site [73,74,75]. Therefore, we inquired whether *APEX1* knockout cells can repair uracil in DNA using our reporter system. In this case, A is expected to be incorporated opposite to the unrepaired lesion and produce the fluorescent EGFP Q205K variant (Figure 1a). U, sU, and sUs were all efficiently processed by human UNG in vitro, while AP sites generated from sU and sUs were alkali-labile but much more resistant to APE1 than the natural AP site (Figure 4a,b). Again, wild-type cells showed no difference between the fluorescence levels when transfected with the U-construct or the control A-construct (*p* = 0.16), confirming that U is quickly repaired in the cellular context (Figure 4c). One *APEX1* knockout cell line (1C4) showed a statistically significant but moderate (~30%) increase in the population median of EGFP-producing cells, while the difference in the other line (2A9) did not reach significance (*p* = 0.32). The magnitude of the effect of the *APEX1* knockout was comparable with the effect on the F repair.

When we replaced U with sU, the population of fluorescent cells increased only ~1.8-fold for both wild-type and knockout cells (Figure 4c). This was in stark contrast to the more than an order of magnitude increase caused by the phosphorothioate group of the F residue. Apparently, the natural AP site formed after U excision can undergo repair with DNA nicking by β-elimination, a reaction impossible for the F abasic site. Since wild-type and knockout cells were affected equally, any modest repair impediment resulting from the 5′-phosphate replacement with 5′-phosphorothioate is not dependent on APE1.

AP endonucleases do not cleave the 3′-phosphodiester bond of the AP site; instead, the 3′-phosphate can be eliminated by DNA glycosylases with an AP lyase activity, of which human cells possess five: NTHL1, OGG1, NEIL1, NEIL2, and NEIL3. To test the hypothesis that the residual repair depends on one of the cellular AP lyases, we have introduced the second phosphorothioate substitution 3′ to the U lesion. This modification caused a significant increase in the population of fluorescent cells in all three cell lines (Figure 4c). Compared with the U-construct, the fluorescence increased eightfold for the wild-type cells, sixfold for the 1C4 cells, and fivefold for the 2A9 cells, while in comparison with the sU-construct, the increase was 4.4-fold, 3.5-fold, and 2.8-fold, respectively. In 2A9 cells, the effect was significantly lower than in WT and 1C4 cells, but the pooled data from both *APEX1* knockout lines were not statistically different from the wild type. This indicates that AP lyases could possibly participate in the repair of U in human cells if the hydrolysis of the 5′-phosphodiester bond is blocked.

### 2.5. NTHL1 Contributes to the Backup Uracil Repair

**Figure 5:**
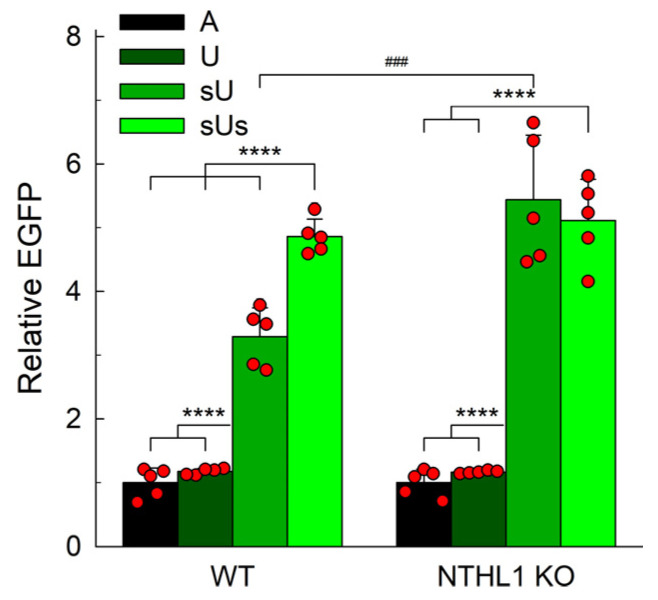
Uracil-induced transcriptional mutagenesis in wild-type HeLa cells and its *NTHL1^KO^* derivative. Relative EGFP expression normalized for the fluorescence of the control A-construct (*n* = 5, mean ± SD shown). Differences between constructs: *p* < 0.001 (****). Differences between genotypes: *p* < 0.005 (###).

Bifunctional DNA glycosylases possess an AP lyase activity and thus can contribute to the processing of natural abasic sites generated at the first step of uracil repair. Five bifunctional DNA glycosylases, NTHL1, OGG1, NEIL1, NEIL2, and NEIL3, are known in human cells. Of those, OGG1 has only a weak lyase activity [76,77,78,79]. NEIL1 and NEIL2 can process AP sites and bypass a requirement for APE1 [45,80]. However, these proteins belong to the helix–two-turn–helix superfamily whose members catalyze concerted elimination of 3′- and 5′-phosphates [81,82] and are therefore poorly suited for the efficient repair of sU (Section 2.4). NTHL1, on the other hand, is an efficient AP lyase that catalyzes β-elimination at pre-formed AP sites [83,84] but was not studied before as a possible AP site-processing enzyme in vivo. We thus addressed U repair in HeLa NTHL1 knockout cells described recently [85]. No significant difference in the repair of U or sUs between wild-type and mutant cells was found (Figure 5). However, when transfected with the sU-constructs, the NTHL1 knockout cells demonstrated a 4.7-fold increase in the fluorescent population compared to the U-construct. This was significantly higher than the 2.8-fold increase observed in wild-type cells. Moreover, whereas the wild-type cells produced an additional 1.5-fold increase with the sUs-construct, the fluorescent population of the knockout cells remained at the same level for both sU and sUs repair. Apparently, even if the miscoding U is removed, AP sites persisting in the absence of NTHL1 still give rise to fluorescent EGFP. Thus, at least part of the sU repair observed in wild-type cells likely depends on NTHL1.

## 3. Discussion

AP sites continuously appear in DNA through spontaneous depurination and as the first reaction intermediate of base excision repair. Due to their inability to form canonical base pairs and their chemical instability, AP sites are cytotoxic and highly mutagenic and have to be repaired quickly. In humans, the main repair enzyme for these lesions is APE1, an endonuclease that specifically hydrolyzes the phosphodiester bond 5′ of an AP site [9,10]. The resulting nick is further processed via the BER pathway [5,6,7]. In addition, a number of human enzymes have been reported to cleave AP sites in vitro, catalyzing either hydrolysis of the 5′-phosphodiester bond (TDP1, APE2, APLF, TATDN1, TATDN3) [43,44,46,47] or elimination of the 3′-phosphate (the AP lyase activity of NTHL1, OGG1, and NEIL1–NEIL3 DNA glycosylases) [40,41]. However, the possibility of AP site repair through the action of these enzymes in living cells is still unclear.

Here, we address the alternatives for the APE1-initiated repair of AP sites, making use of *APEX1* knockout cells generated by us earlier from the human HEK293FT line [39]. *APEX1* depletion in vertebrates is lethal at the embryonic or early postnatal stages [28,29,30,31,32,34], but complete or conditional knockouts in adult organisms and in cell lines can be tolerated [33,35,36,37,38,39]. The 1C4 and 2A9 *APEX1^KO^* monoclonal lines used here demonstrate normal morphology, doubling time, and cell cycle but bear no wild-type *APEX1* alleles, show increased sensitivity to MMS, have a higher background level of natural AP sites, lack detectable immunoreactive APE1 protein, and their extracts do not cleave oligonucleotides containing a natural AP site or its F analog [39]. Thus, they appear to be well-suited to study the fate of AP sites in living cells in the absence of APE1. Despite being similar overall, the lines show slight differences in their behavior: for instance, 2A9 cells have fewer background AP sites but are somewhat more sensitive to H_2_O_2_ than 1C4 cells [39]. As this variance likely reflects non-identical life trajectories from a single cell to the monoclonal establishment, in the experiments described here, we analyzed both individual lines and the pooled data for the two lines, when applicable.

To compare the repair of various DNA lesions in cells with different genetic backgrounds, we used an assay based on the restoration of fluorescence of the non-functional EGFP reporter as a result of NMP misincorporation by an RNA polymerase, also known as “transcriptional mutagenesis”. The *eGFP* coding sequence conveniently has two nickase sites near each other, making it possible to introduce any lesion between them by oligonucleotide replacement [57]. Several nonsense and missense mutations in this region that result in non-fluorescent EGFP have been identified [51,57,61]. In the presence of DNA damage, repair converts the *eGFP* sequence back to coding the non-fluorescent form; on the contrary, lack of repair is manifested in the appearance of fluorescing cells. The assay was successfully applied to analyze the repair of 8-oxoG, U, thymine glycol, AP sites, and single-strand breaks in cellulo [51,61,65,86,87,88,89]. Since the TM outcome is strongly influenced by the ability of RNA polymerase II to bypass the lesion and by the accompanying misincorporation spectrum, the TM assay is best suited for quantitative comparisons of the same lesions under the conditions of different repair capacities, which can be modulated either by the host cell genotype or by small lesion modifications affecting their removal by DNA repair. The latter option has been exploited by the replacement of internucleoside phosphates with phosphorothioates, which are much more resistant to hydrolysis and β-elimination [51,61,65,90]. As expected, wild-type cells efficiently repaired F and U, apparently via the BER pathway, as evidenced by low fluorescence of the cells transfected with the respective constructs and its prominent increase upon hindering BER with a 5′-phosphorothioate substitution. The 5′-phosphorothioate group is enantiomeric; APE1 cleaves the *R*_p_ isomer ~20-fold slower than the phosphate linkage and does not detectably cleave the *S*_p_ isomer [62,63]. Chemical synthesis produces a racemic mixture, which is mostly used without further separation in most practical applications employing phosphorothioate to block nuclease activity [91]. A mixture of isomers was also used here, so one can roughly estimate the ability of cellular APE1 to process such substrates as ~1/40 of the all-phosphate constructs.

Given the absence of an appreciable F-cleaving activity in both *APEX1^KO^* cell lines, we were surprised to see little difference from the wild-type parental line in the repair of this AP site analog. So far, F was regarded as a typical lesion repaired by the long-patch BER branch initiated by APE1 [92,93]. Low fluorescence of F-construct-transfected cells cannot be explained by poor RNA polymerase bypass of the non-instructive lesion since, with an equally non-instructive sF, the fluorescence population median increased 28–44-fold in both wild-type and knockout cells. The nature of the back-up activity remains murky at present. Since F is resistant to β-elimination and sF is repaired much worse, the enzyme responsible for the back-up F repair must hydrolyze the 5′-phosphodiester bond. We have ruled out APE2 as a candidate by making double *APEX1 APEX2* knockouts and finding no significant difference from the single *APEX1^KO^* genotype. Recently, it has been shown that sF (and, by inference, F) is partly removed by NER since its repair is suppressed in *XPA*-, *XPC*-, and *XPF*-negative cells [51]. However, NER would not make a distinction between F and sF and is unlikely to explain the 5′-phosphorothioate-sensitive F repair. Other enzymes with 5′-endonuclease activities, such as TDP1, APLF, TATDN1, and TATDN3, or more general DNases, can also be considered but will require further investigation. Apparently, the observed lack of AP site processing via these alternative pathways by cell extracts ([39] and Supplementary Figure S1) is due to their lower repair capacity and/or suboptimal activity under the conditions optimized to detect cleavage by APE1.

As in the case with F, APE1-deficient cells were indistinguishable from wild-type HEK293FT cells in their ability to repair U. All four human DNA glycosylases that remove U from DNA (UNG, TDG, SMUG1, and MBD4) are monofunctional and yield a natural (aldehydic) AP site as their reaction product. Due to their instability, natural AP sites cannot be introduced into reporter plasmid constructs directly since they are prone to elimination under the conditions of cell transfection (D.V.K., A.V.Y., and D.O.Z., unpublished observations). When produced from U or other damaged nucleotides *in situ*, natural AP sites are normally processed by APE1. However, unlike for F, the option of catalyzed or spontaneous β-elimination is also available for them. Consistent with this, we see a much less pronounced effect of an sU substitution, which still can be nicked at the 3′-side, in comparison with the sF vs F repair. Only when β-elimination is impeded by an additional 3′-phosphorothioate substitution does the fluorescence of the EGFP reporter substantially increase. Knockout of *NTHL1* eliminates the difference between sU and sUs repair in wild-type cells, suggesting that NTHL1 DNA glycosylase/AP lyase is responsible, at least in part, for the nicking if the APE1 activity is blocked. A highly relevant question is how the 3′-PUA left after NTHL1 is processed if it bears a 5′-phosphorothioate group resistant to hydrolysis and elimination. One interesting possibility is that BER, in this case, may proceed along its recently discovered 5′-gap branch, in which the RECQ1 helicase unwinds several base pairs 5′ of a nick, XPF–ERCC1 endonuclease excises the formed 3′-terminal flap, and the reaction is then follows the long-patch BER pathway [94]. The problem of end cleaning is thus circumvented.

Employing AP lyases to bypass the requirement for an AP endonuclease activity in BER is not without precedent. The best-studied example is the APE1-independent, PNKP-dependent BER branch that operates when the repair is initiated by NEIL1 or NEIL2 [45,80]. Although these glycosylases recognize oxidized pyrimidines and catalyze β,δ-elimination, leaving a 3′-terminal phosphate, they can also use pre-formed AP sites and process them in the same manner. In particular, there is evidence that NEIL1 and NEIL2 bind TDG and promote the cleavage of AP sites formed from 5-formulcytosine and 5-carboxylcytosine during active demethylation [95,96]. In *Arabidopsis*, AP sites formed by glycosylases are apparently nicked by AP endonucleases, while those originating from spontaneous depurination are processed by MMH, a homolog of human NEIL proteins [97]. In fission yeast, *Schizosaccharomyces pombe*, Nth1 is established as the main AP site-processing enzyme while the AP endonuclease homologs Apn1 and Apn2 participate in 3′-end cleaning [98,99,100,101]. Hence, NTHL1 or other AP lyases are well suited to serve as back-up enzymes for BER initiation in human cells.

One possible limitation of this study is the possibility of the reporter plasmid replication in HEK293FT cells since they were created by transformation with SV40 T antigen, and the pZAJ vectors carry the SV40 replication origin. Replication may influence the TM-dependent fluorescent outcome in both directions, either triggering error-free or error-prone lesion repair or promoting mutations, which makes its effects hard to predict. To alleviate this potential source of uncertainty, we measured the fluorescence 24 h post-transfection, which minimizes the number of replication cycles while allowing enough time for EGFP production. At this time point, we saw no difference in the EGFP fluorescence when wild-type pZAJ-5c cells transfected into HEK293FT and HEK293T cells that contain T antigen and HEK293A that do not. The interplay between DNA repair, DNA replication, and transcriptional mutagenesis in reporter plasmids merits special investigation.

## 4. Materials and Methods

### 4.1. Enzymes, Oligonucleotides, Plasmids, and Cells

Nb.Bpu10I and Nt.Bpu10I nickases, human APE1, and *E. coli* Ung and Nfo were from New England Biolabs (Ipswich, MA, USA), phage T4 DNA ligase and polynucleotide kinase, from Thermo Fisher Scientific (Waltham, MA, USA). Human UNG was purified as described [102]. HEK293FT *APEX1^KO^* cells 1C4 and 2A9 had been generated and described earlier [39] and were from the laboratory stocks. HeLa *NTHL1* knockout cells [85] and pZAJ_Q205* plasmid [61] were a kind gift from Dr. Andriy Khobta (Friedrich Schiller University Jena, Germany). Oligonucleotides (Table 1) were synthesized in-house from commercially available phosphoramidites (Glen Research, Sterling, VA, USA). To make the racemic phosphorothioate substitution, 3-((dimethylaminomethylidene)amino)-3*H*-1,2,4-dithiazole-3-thione (DDTT; Glen Research) was used. The standard oxidation step in solid phase synthesis was replaced with the treatment of the phosphite-triester link with DDTT solution in pyridine/acetonitrile (6:4, *v*/*v*) for 1 min. The final product was cleaved from the solid support with ammonia/methylamine solution (1:1) at room temperature for 1 day. The oligonucleotides were purified by reverse phase HPLC on a Zorbax SB-C18 5 mm column using an Agilent1200 system (Santa Clara, CA, USA).

### 4.2. Enzyme Assays

For enzyme assays, ts.613 series oligonucleotides labeled with ^32^P were annealed with an equimolar amount of Q205_compl. Cleavage of U-, sU-, and sUs-containing duplexes by human UNG was performed in 25 mM Tris–HCl (pH 7.5), 50 mM NaCl, 1 mM EDTA, 1 mM DTT, 50 nM substrate, 100 nM UNG at 37 °C for 10 min. The reactions were quenched with NaOH (0.1 M final), heated at 95 °C for 2 min, and neutralized with HCl. Cleavage of AP sites generated from U-, sU-, and sUs-containing duplexes was performed in 50 mM Tris–HCl (pH 7.5), 100 mM NaCl, 5 mM MgCl_2_, 1 mM DTT, 50 nM substrate, 2 U *E. coli* Ung and 0.1 U APE1 at 37 °C for 10 min. The reactions were terminated either as above to follow AP site formation or by adding formamide to 40% and heating at 95 °C for 1 min to follow AP site cleavage. In all cases, the products were resolved by electrophoresis in 20% polyacrylamide/7.2 M urea and visualized using a Typhoon FLA 9500 system (Danaher, Washington, DC, USA).

### 4.3. Generation of APEX1^KO^ APEX2^KO^ Cells

To establish *APEX2* knockout cell lines, a standard workflow based on cell transformation with a plasmid carrying both Cas9 and sgRNA was used [103]. sgRNA targeted a protospacer close to the start of the *APEX2* coding sequence (Supplementary Figure S2a). *APEX1* knockout 1C4 cells [39] were transfected with the pX458 plasmid carrying the sgRNA sequence (Table 1). Twenty-four hours post-transfection, the cells were sorted based on the EGFP fluorescence and diluted to single cells to generate monoclones. The overall editing efficiency was estimated by sequence trace decomposition (TIDE) [104] (Supplementary Figure S2b). Six clones were obtained, sequenced, and analyzed by TIDE, and two clones (12KO1 and 12KO2) that contained frameshift mutations were picked for further analysis (Supplementary Figure S2c–f). To characterize the mutations in the target region of the *APEX2* gene, 13 subclones obtained by TA-cloning (TA Cloning Kit, Thermo Fisher Scientific) were sequenced from each clone (Supplementary Figure S2g,h). Both clones carried mutations generating the protein missing most of the catalytic domain (Supplementary Figure S2i). For real-time RT-PCR, total RNA was extracted using the RUplus kit (Biolabmix, Russia) and treated with Dnase I (Thermo Fisher Scientific). One µg total RNA was reverse transcribed with the M-MuLV –RH kit and oligo(dT)_16_ primer (Biolabmix). Real-time PCR was performed using the BioMaster HS-qPCR SYBR Blue kit (Biolabmix) on a CFX Opus Real-time PCR System (Bio-Rad, Hercules, CA, USA) with the following program: 5 min at 95 °C, then 35 cycles of 10 s at 95 °C, 30 s at 62.7 °C. The primers are listed in Table 1; *GAPDH* mRNA served as a reference.

### 4.4. Damaged Plasmids

Plasmid vector pZAJ_Q205* was used to generate control and damaged constructs, as described previously [57] (Supplementary Figure S3a). The reaction mixture (250 µL) contained 50 µg of plasmid DNA, 10 mM Tris–HCl (pH 8.5), 10 mM MgCl_2_, 100 mM KCl, 0.1 mg/mL BSA and 50 U of Nb.Bpu10I or Nt.Bpu10I. The reaction was allowed to proceed for 2 h at 37 °C followed by enzyme inactivation for 20 min at 80 °C. To confirm the presence of two nicks, 100 ng of the treated plasmid and a 180-fold molar excess of the competitor oligonucleotide Q205_compl (Table 1) with a non-phosphorylated 5′-end were heated at 80 °C for 10 min in T4 DNA ligase buffer (Thermo Fisher Scientific), cooled to 4 °C, supplemented with 2 U of T4 DNA ligase and incubated for 1 h at 37 °C. A parallel reaction without the competitor oligonucleotide served as a ligation control. After enzyme inactivation at 65 °C for 15 min, the reaction products were analyzed by agarose gel electrophoresis. Complete suppression of ligation by the competitor indicates the formation of two nicks (Supplementary Figure S3b,c). The double-nicked plasmids were converted to gapped ones by heating and cooling under the same conditions but with a 900-fold molar excess of the competitor. The excess oligonucleotides were removed by ultrafiltration using an Amicon Ultracel 20 centrifugal filter unit (MilliporeSigma, Burlington, MA, USA), and gap formation was confirmed by analytical ligation with non-phosphorylated ts613_A oligo (Supplementary Figure S3d,e). To ligate a damaged oligonucleotide into the gap, 20 µg of the gapped plasmid was incubated with a 180-fold molar excess of the oligonucleotide (ts.613 series, Table 1) and 250 U of T4 polynucleotide kinase for 30 min at 37 °C, and the enzyme was inactivated at 80 °C for 10 min. Then 100 U of T4 DNA ligase was added to the reaction mixture on ice, and the mixture was incubated for 1 h at 37 °C followed by 15 min at 65 °C. An aliquot was analyzed by electrophoresis with a control sample, which was incubated without T4 polynucleotide kinase, to confirm successful ligation. The constructs were purified from the excess oligonucleotide by ultrafiltration, as described above. To confirm the presence of a lesion, 100 ng of the resulting construct was treated for 30 min at 37 °C with a large excess of APE1 (4 U) or *E. coli* Ung (2 U) and Nfo (2 U), inactivated for 20 min at 65 °C, and analyzed by electrophoresis (Supplementary Figure S3f,g).

### 4.5. Transcriptional Mutagenesis Experiments

Cells were seeded at 0.3 × 10^6^ per well in a six-well plate. After 24 h, the cells were transfected with a mixture of 400 ng of the EGFP-based construct and 400 ng of pDsRed-Monomer-N1 (TaKaRa Bio, Kyoto, Japan) using the Effectene reagent (Qiagen, Venlo, The Netherlands). After another 24 h, the cells were trypsinized, collected, fixed in 1% formaldehyde, and analyzed on a CytoFlex flow cytometer (Beckman Coulter, Brea, CA, USA). To assess the relative level of EGFP expression, only transfected cells were taken into account based on the level of DsRed fluorescence. The median fluorescence of the EGFP-positive, DsRed-positive cells was normalized for the median fluorescence of the pZAJ-5c construct encoding a fully functional EGFP protein [51,86,87]. To control the variation in the non-specific fluorescence, these values were then normalized for the median fluorescence of the cells transfected with the A-construct. Statistical significance was calculated using a two-tailed Student’s *t*-test from three to five biological replicates. The Bonferroni correction for multiple comparisons was applied.

## Figures and Tables

**Table 1:** Oligonucleotides used in this work.

Oligo ID	Sequence (5′→3′)	Modification
Construction of lesion-containing plasmids		
ts.613_A	TCAGGGCGGACTAGGTGC	
ts.613_F	TCAGGGCGGACTXGGTGC	X = F
ts.613_sF	TCAGGGCGGACTXGGTGC	X = sF
ts.613_U	TCAGGGCGGACTXGGTGC	X = U
ts.613_sU	TCAGGGCGGACTXGGTGC	X = sU
ts.613_sUs	TCAGGGCGGACTXGGTGC	X = sUs
Q205_compl	GCACCTAGTCCGCCCTGA	
sgRNA cloning for *APEX2* knockout		
APEX2_top	CACCGATTCGGAGACCCCTGCAAG	
APEX2_bot	AAACCTTGCAGGGGTCTCCGAATC	
TA-cloning and TIDE for *APEX2* knockout		
APEX2_fwd1	AGGAAGCAGTTCGCTCGC	
APEX2_rev1	CTGAGGGGAGATAAGAGGGTGAA	
APEX2_fwd2	CTTTGCTTCCTTCAGCGTCC	
APEX2_rev2	TTCGGGGGTTTGACTTGG	
Real-time RT-PCR for *APEX2* mRNA		
APEX2_rt_fwd	CTGGAACATCAATGGGATTCGG	
APEX2_rt_rev	CCAGCTCGTCCAAAATGCG	
GAPDH_rt_fwd	ACATCGCTCAGACACCAT	
GAPDH_rt_rev	TGTAGTTGAGGTCAATGAAGG	

## Data Availability

All data are reported in the paper and the Supplementary.

## Supplementary Materials

The following supporting information can be downloaded at https://www.mdpi.com/article/10.3390/ijms25010064/s1.

## References

[1] Lindahl T., Nyberg B. (1972). Rate of depurination of native deoxyribonucleic acid. Biochemistry.

[2] Atamna H., Cheung I., Ames B.N. (2000). A method for detecting abasic sites in living cells: Age-dependent changes in base excision repair. Proc. Natl. Acad. Sci. USA.

[3] Boiteux S., Guillet M. (2004). Abasic sites in DNA: Repair and biological consequences in *Saccharomyces cerevisiae*. DNA Repair.

[4] Thompson P.S., Cortez D. (2020). New insights into abasic site repair and tolerance. DNA Repair.

[5] Zharkov D.O. (2008). Base excision DNA repair. Cell. Mol. Life Sci..

[6] Beard W.A., Horton J.K., Prasad R., Wilson S.H. (2019). Eukaryotic base excision repair: New approaches shine light on mechanism. Annu. Rev. Biochem..

[7] Wozniak K.J., Simmons L.A. (2022). Bacterial DNA excision repair pathways. Nat. Rev. Microbiol..

[8] Fortini P., Dogliotti E. (2007). Base damage and single-strand break repair: Mechanisms and functional significance of short- and long-patch repair subpathways. DNA Repair.

[9] Tell G., Fantini D., Quadrifoglio F. (2010). Understanding different functions of mammalian AP endonuclease (APE1) as a promising tool for cancer treatment. Cell. Mol. Life Sci..

[10] Whitaker A.M., Freudenthal B.D. (2018). APE1: A skilled nucleic acid surgeon. DNA Repair.

[11] Xanthoudakis S., Miao G., Wang F., Pan Y.-C.E., Curran T. (1992). Redox activation of Fos–Jun DNA binding activity is mediated by a DNA repair enzyme. EMBO J..

[12] Kuninger D.T., Izumi T., Papaconstantinou J., Mitra S. (2002). Human AP-endonuclease 1 and hnRNP-L interact with a nCaRE-like repressor element in the AP-endonuclease 1 promoter. Nucleic Acids Res..

[13] Bhakat K.K., Izumi T., Yang S.-H., Hazra T.K., Mitra S. (2003). Role of acetylated human AP-endonuclease (APE1/Ref-1) in regulation of the parathyroid hormone gene. EMBO J..

[14] Hanson S., Kim E., Deppert W. (2005). Redox factor 1 (Ref-1) enhances specific DNA binding of p53 by promoting p53 tetramerization. Oncogene.

[15] Seemann S., Hainaut P. (2005). Roles of thioredoxin reductase 1 and APE/Ref-1 in the control of basal p53 stability and activity. Oncogene.

[16] Sengupta S., Mantha A.K., Mitra S., Bhakat K.K. (2011). Human AP endonuclease (APE1/Ref-1) and its acetylation regulate YB-1-p300 recruitment and RNA polymerase II loading in the drug-induced activation of multidrug resistance gene *MDR1*. Oncogene.

[17] Hajkova P., Jeffries S.J., Lee C., Miller N., Jackson S.P., Surani M.A. (2010). Genome-wide reprogramming in the mouse germ line entails the base excision repair pathway. Science.

[18] Weber A.R., Krawczyk C., Robertson A.B., Kuśnierczyk A., Vågbø C.B., Schuermann D., Klungland A., Schär P. (2016). Biochemical reconstitution of TET1–TDG–BER-dependent active DNA demethylation reveals a highly coordinated mechanism. Nat. Commun..

[19] Yamamori T., DeRicco J., Naqvi A., Hoffman T.A., Mattagajasingh I., Kasuno K., Jung S.-B., Kim C.-S., Irani K. (2010). SIRT1 deacetylates APE1 and regulates cellular base excision repair. Nucleic Acids Res..

[20] Hwang B.-J., Jin J., Gao Y., Shi G., Madabushi A., Yan A., Guan X., Zalzman M., Nakajima S., Lan L. (2015). SIRT6 protein deacetylase interacts with MYH DNA glycosylase, APE1 endonuclease, and Rad9–Rad1–Hus1 checkpoint clamp. BMC Mol. Biol..

[21] Madlener S., Ströbel T., Vose S., Saydam O., Price B.D., Demple B., Saydam N. (2013). Essential role for mammalian apurinic/apyrimidinic (AP) endonuclease Ape1/Ref-1 in telomere maintenance. Proc. Natl. Acad. Sci. USA.

[22] Li M., Yang X., Lu X., Dai N., Zhang S., Cheng Y., Zhang L., Yang Y., Liu Y., Yang Z. (2018). APE1 deficiency promotes cellular senescence and premature aging features. Nucleic Acids Res..

[23] Vascotto C., Fantini D., Romanello M., Cesaratto L., Deganuto M., Leonardi A., Radicella J.P., Kelley M.R., D’Ambrosio C., Scaloni A. (2009). APE1/Ref-1 interacts with NPM1 within nucleoli and plays a role in the rRNA quality control process. Mol. Cell. Biol..

[24] Malfatti M.C., Balachander S., Antoniali G., Koh K.D., Saint-Pierre C., Gasparutto D., Chon H., Crouch R.J., Storici F., Tell G. (2017). Abasic and oxidized ribonucleotides embedded in DNA are processed by human APE1 and not by RNase H2. Nucleic Acids Res..

[25] Barnes T., Kim W.-C., Mantha A.K., Kim S.-E., Izumi T., Mitra S., Lee C.H. (2009). Identification of Apurinic/apyrimidinic endonuclease 1 (APE1) as the endoribonuclease that cleaves c-*myc* mRNA. Nucleic Acids Res..

[26] Antoniali G., Serra F., Lirussi L., Tanaka M., D’Ambrosio C., Zhang S., Radovic S., Dalla E., Ciani Y., Scaloni A. (2017). Mammalian APE1 controls miRNA processing and its interactome is linked to cancer RNA metabolism. Nat. Commun..

[27] Fan Z., Beresford P.J., Zhang D., Xu Z., Novina C.D., Yoshida A., Pommier Y., Lieberman J. (2003). Cleaving the oxidative repair protein Ape1 enhances cell death mediated by granzyme A. Nat. Immunol..

[28] Xanthoudakis S., Smeyne R.J., Wallace J.D., Curran T. (1996). The redox/DNA repair protein, Ref-1, is essential for early embryonic development in mice. Proc. Natl. Acad. Sci. USA.

[29] Ludwig D.L., MacInnes M.A., Takiguchi Y., Purtymun P.E., Henrie M., Flannery M., Meneses J., Pedersen R.A., Chen D.J. (1998). A murine AP-endonuclease gene-targeted deficiency with post-implantation embryonic progression and ionizing radiation sensitivity. Mutat. Res..

[30] Meira L.B., Devaraj S., Kisby G.E., Burns D.K., Daniel R.L., Hammer R.E., Grundy S., Jialal I., Friedberg E.C. (2001). Heterozygosity for the mouse *Apex* gene results in phenotypes associated with oxidative stress. Cancer Res..

[31] Wang Y., Shupenko C.C., Melo L.F., Strauss P.R. (2006). DNA repair protein involved in heart and blood development. Mol. Cell. Biol..

[32] Han D., Schomacher L., Schüle K.M., Mallick M., Musheev M.U., Karaulanov E., Krebs L., von Seggern A., Niehrs C. (2019). NEIL1 and NEIL2 DNA glycosylases protect neural crest development against mitochondrial oxidative stress. eLife.

[33] Stetler R.A., Gao Y., Leak R.K., Weng Z., Shi Y., Zhang L., Pu H., Zhang F., Hu X., Hassan S. (2016). APE1/Ref-1 facilitates recovery of gray and white matter and neurological function after mild stroke injury. Proc. Natl. Acad. Sci. USA.

[34] Dumitrache L.C., Shimada M., Downing S.M., Kwak Y.D., Li Y., Illuzzi J.L., Russell H.R., Wilson D.M., McKinnon P.J. (2018). Apurinic endonuclease-1 preserves neural genome integrity to maintain homeostasis and thermoregulation and prevent brain tumors. Proc. Natl. Acad. Sci. USA.

[35] Masani S., Han L., Yu K. (2013). Apurinic/apyrimidinic endonuclease 1 is the essential nuclease during immunoglobulin class switch recombination. Mol. Cell. Biol..

[36] Chen T., Liu C., Lu H., Yin M., Shao C., Hu X., Wu J., Wang Y. (2017). The expression of APE1 in triple-negative breast cancer and its effect on drug sensitivity of olaparib. Tumor Biol..

[37] Izumi T., Brown D.B., Naidu C.V., Bhakat K.K., MacInnes M.A., Saito H., Chen D.J., Mitra S. (2005). Two essential but distinct functions of the mammalian abasic endonuclease. Proc. Natl. Acad. Sci. USA.

[38] Malfatti M.C., Gerratana L., Dalla E., Isola M., Damante G., Di Loreto C., Puglisi F., Tell G. (2019). APE1 and NPM1 protect cancer cells from platinum compounds cytotoxicity and their expression pattern has a prognostic value in TNBC. J. Exp. Clin. Cancer Res..

[39] Kim D.V., Kulishova L.M., Torgasheva N.A., Melentyev V.S., Dianov G.L., Medvedev S.P., Zakian S.M., Zharkov D.O. (2021). Mild phenotype of knockouts of the major apurinic/apyrimidinic endonuclease APEX1 in a non-cancer human cell line. PLoS ONE.

[40] Stivers J.T., Jiang Y.L. (2003). A mechanistic perspective on the chemistry of DNA repair glycosylases. Chem. Rev..

[41] Dizdaroglu M., Coskun E., Jaruga P. (2017). Repair of oxidatively induced DNA damage by DNA glycosylases: Mechanisms of action, substrate specificities and excision kinetics. Mutat. Res..

[42] Pommier Y., Huang S.-y.N., Gao R., Das B.B., Murai J., Marchand C. (2014). Tyrosyl-DNA-phosphodiesterases (TDP1 and TDP2). DNA Repair.

[43] Burkovics P., Szukacsov V., Unk I., Haracska L. (2006). Human Ape2 protein has a 3′–5′ exonuclease activity that acts preferentially on mismatched base pairs. Nucleic Acids Res..

[44] Lebedeva N.A., Rechkunova N.I., El-Khamisy S.F., Lavrik O.I. (2012). Tyrosyl-DNA phosphodiesterase 1 initiates repair of apurinic/apyrimidinic site. Biochimie.

[45] Wiederhold L., Leppard J.B., Kedar P., Karimi-Busheri F., Rasouli-Nia A., Weinfeld M., Tomkinson A.E., Izumi T., Prasad R., Wilson S.H. (2004). AP endonuclease-independent DNA base excision repair in human cells. Mol. Cell.

[46] Kanno S.-I., Kuzuoka H., Sasao S., Hong Z., Lan L., Nakajima S., Yasui A. (2007). A novel human AP endonuclease with conserved zinc-finger-like motifs involved in DNA strand break responses. EMBO J..

[47] Dorival J., Eichman B.F. (2023). Human and bacterial TatD enzymes exhibit apurinic/apyrimidinic (AP) endonuclease activity. Nucleic Acids Res..

[48] Huang J.-C., Hsu D.S., Kazantsev A., Sancar A. (1994). Substrate spectrum of human excinuclease: Repair of abasic sites, methylated bases, mismatches, and bulky adducts. Proc. Natl. Acad. Sci. USA.

[49] Torres-Ramos C.A., Johnson R.E., Prakash L., Prakash S. (2000). Evidence for the involvement of nucleotide excision repair in the removal of abasic sites in yeast. Mol. Cell. Biol..

[50] Kim N., Jinks-Robertson S. (2010). Abasic sites in the transcribed strand of yeast DNA are removed by transcription-coupled nucleotide excision repair. Mol. Cell. Biol..

[51] Kitsera N., Rodriguez-Alvarez M., Emmert S., Carell T., Khobta A. (2019). Nucleotide excision repair of abasic DNA lesions. Nucleic Acids Res..

[52] Wittschieben B.Ø., Iwai S., Wood R.D. (2005). DDB1-DDB2 (xeroderma pigmentosum group E) protein complex recognizes a cyclobutane pyrimidine dimer, mismatches, apurinic/apyrimidinic sites, and compound lesions in DNA. J. Biol. Chem..

[53] Scrima A., Koníčková R., Czyzewski B.K., Kawasaki Y., Jeffrey P.D., Groisman R., Nakatani Y., Iwai S., Pavletich N.P., Thomä N.H. (2008). Structural basis of UV DNA-damage recognition by the DDB1-DDB2 complex. Cell.

[54] Jang S., Kumar N., Beckwitt E.C., Kong M., Fouquerel E., Rapić-Otrin V., Prasad R., Watkins S.C., Khuu C., Majumdar C. (2019). Damage sensor role of UV-DDB during base excision repair. Nat. Struct. Mol. Biol..

[55] Klein H.L., Bačinskaja G., Che J., Cheblal A., Elango R., Epshtein A., Fitzgerald D.M., Gómez-González B., Khan S.R., Kumar S. (2019). Guidelines for DNA recombination and repair studies: Cellular assays of DNA repair pathways. Microb. Cell.

[56] Owiti N.A., Nagel Z.D., Engelward B.P. (2021). Fluorescence sheds light on DNA damage, DNA repair, and mutations. Trends Cancer.

[57] Lühnsdorf B., Kitsera N., Warken D., Lingg T., Epe B., Khobta A. (2012). Generation of reporter plasmids containing defined base modifications in the DNA strand of choice. Anal. Biochem..

[58] Raetz A.G., Xie Y., Kundu S., Brinkmeyer M.K., Chang C., David S.S. (2012). Cancer-associated variants and a common polymorphism of *MUTYH* exhibit reduced repair of oxidative DNA damage using a GFP-based assay in mammalian cells. Carcinogenesis.

[59] Chaim I.A., Gardner A., Wu J., Iyama T., Wilson D.M., Samson L.D. (2017). A novel role for transcription-coupled nucleotide excision repair for the in vivo repair of 3,*N*^4^-ethenocytosine. Nucleic Acids Res..

[60] Chaim I.A., Nagel Z.D., Jordan J.J., Mazzucato P., Ngo L.P., Samson L.D. (2017). In vivo measurements of interindividual differences in DNA glycosylases and APE1 activities. Proc. Natl. Acad. Sci. USA.

[61] Rodriguez-Alvarez M., Kim D., Khobta A. (2020). EGFP reporters for direct and sensitive detection of mutagenic bypass of DNA lesions. Biomolecules.

[62] Wilson D.M., Takeshita M., Grollman A.P., Demple B. (1995). Incision activity of human apurinic endonuclease (Ape) at abasic site analogs in DNA. J. Biol. Chem..

[63] Mundle S.T., Delaney J.C., Essigmann J.M., Strauss P.R. (2009). Enzymatic mechanism of human apurinic/apyrimidinic endonuclease against a THF AP site model substrate. Biochemistry.

[64] Iwai S., Maeda M., Shirai M., Shimada Y., Osafune T., Murata T., Ohtsuka E. (1995). Reaction mechanism of T4 endonuclease V determined by analysis using modified oligonucleotide duplexes. Biochemistry.

[65] Allgayer J., Kitsera N., Bartelt S., Epe B., Khobta A. (2016). Widespread transcriptional gene inactivation initiated by a repair intermediate of 8-oxoguanine. Nucleic Acids Res..

[66] Li H., Endutkin A.V., Bergonzo C., Fu L., Grollman A.P., Zharkov D.O., Simmerling C. (2017). DNA deformation-coupled recognition of 8-oxoguanine: Conformational kinetic gating in human DNA glycosylase. J. Am. Chem. Soc..

[67] Demple B., Sung J.-S. (2005). Molecular and biological roles of Ape1 protein in mammalian base excision repair. DNA Repair.

[68] McNeill D.R., Whitaker A.M., Stark W.J., Illuzzi J.L., McKinnon P.J., Freudenthal B.D., Wilson D.M. (2020). Functions of the major abasic endonuclease (APE1) in cell viability and genotoxin resistance. Mutagenesis.

[69] Wang W., Walmacq C., Chong J., Kashlev M., Wang D. (2018). Structural basis of transcriptional stalling and bypass of abasic DNA lesion by RNA polymerase II. Proc. Natl. Acad. Sci. USA.

[70] Mengwasser K.E., Adeyemi R.O., Leng Y., Choi M.Y., Clairmont C., D’Andrea A.D., Elledge S.J. (2019). Genetic screens reveal *FEN1* and *APEX2* as *BRCA2* synthetic lethal targets. Mol. Cell.

[71] Álvarez-Quilón A., Wojtaszek J.L., Mathieu M.-C., Patel T., Appel C.D., Hustedt N., Rossi S.E., Wallace B.D., Setiaputra D., Adam S. (2020). Endogenous DNA 3′ blocks are vulnerabilities for BRCA1 and BRCA2 deficiency and are reversed by the APE2 nuclease. Mol. Cell.

[72] Visnes T., Doseth B., Pettersen H.S., Hagen L., Sousa M.M.L., Akbari M., Otterlei M., Kavli B., Slupphaug G., Krokan H.E. (2009). Uracil in DNA and its processing by different DNA glycosylases. Philos. Trans. R. Soc. B Biol. Sci..

[73] Kubota Y., Nash R.A., Klungland A., Schär P., Barnes D.E., Lindahl T. (1996). Reconstitution of DNA base excision-repair with purified human proteins: Interaction between DNA polymerase β and the XRCC1 protein. EMBO J..

[74] Nicholl I.D., Nealon K., Kenny M.K. (1997). Reconstitution of human base excision repair with purified proteins. Biochemistry.

[75] Akbari M., Otterlei M., Peña-Diaz J., Aas P.A., Kavli B., Liabakk N.B., Hagen L., Imai K., Durandy A., Slupphaug G. (2004). Repair of U/G and U/A in DNA by UNG2-associated repair complexes takes place predominantly by short-patch repair both in proliferating and growth-arrested cells. Nucleic Acids Res..

[76] Zharkov D.O., Rosenquist T.A., Gerchman S.E., Grollman A.P. (2000). Substrate specificity and reaction mechanism of murine 8-oxoguanine-DNA glycosylase. J. Biol. Chem..

[77] Hill J.W., Hazra T.K., Izumi T., Mitra S. (2001). Stimulation of human 8-oxoguanine-DNA glycosylase by AP-endonuclease: Potential coordination of the initial steps in base excision repair. Nucleic Acids Res..

[78] Saitoh T., Shinmura K., Yamaguchi S., Tani M., Seki S., Murakami H., Nojima Y., Yokota J. (2001). Enhancement of OGG1 protein AP lyase activity by increase of APEX protein. Mutat. Res..

[79] Vidal A.E., Hickson I.D., Boiteux S., Radicella J.P. (2001). Mechanism of stimulation of the DNA glycosylase activity of hOGG1 by the major human AP endonuclease: Bypass of the AP lyase activity step. Nucleic Acids Res..

[80] Das A., Wiederhold L., Leppard J.B., Kedar P., Prasad R., Wang H., Boldogh I., Karimi-Busheri F., Weinfeld M., Tomkinson A.E. (2006). NEIL2-initiated, APE-independent repair of oxidized bases in DNA: Evidence for a repair complex in human cells. DNA Repair.

[81] Zharkov D.O., Shoham G., Grollman A.P. (2003). Structural characterization of the Fpg family of DNA glycosylases. DNA Repair.

[82] Kuznetsov N.A., Zharkov D.O., Koval V.V., Buckle M., Fedorova O.S. (2009). Reversible chemical step and rate-limiting enzyme regeneration in the reaction catalyzed by formamidopyrimidine-DNA-glycosylase. Biochemistry.

[83] Aspinwall R., Rothwell D.G., Roldan-Arjona T., Anselmino C., Ward C.J., Cheadle J.P., Sampson J.R., Lindahl T., Harris P.C., Hickson I.D. (1997). Cloning and characterization of a functional human homolog of *Escherichia coli* endonuclease III. Proc. Natl. Acad. Sci. USA.

[84] Ikeda S., Biswas T., Roy R., Izumi T., Boldogh I., Kurosky A., Sarker A.H., Seki S., Mitra S. (1998). Purification and characterization of human NTH1, a homolog of *Escherichia coli* endonuclease III: Direct identification of Lys-212 as the active nucleophilic residue. J. Biol. Chem..

[85] Sarmini L., Meabed M., Emmanouil E., Atsaves G., Robeska E., Karwowski B.T., Campalans A., Gimisis T., Khobta A. (2023). Requirement of transcription-coupled nucleotide excision repair for the removal of a specific type of oxidatively induced DNA damage. Nucleic Acids Res..

[86] Khobta A., Lingg T., Schulz I., Warken D., Kitsera N., Epe B. (2010). Mouse CSB protein is important for gene expression in the presence of a single-strand break in the non-transcribed DNA strand. DNA Repair.

[87] Kitsera N., Stathis D., Lühnsdorf B., Müller H., Carell T., Epe B., Khobta A. (2011). 8-Oxo-7,8-dihydroguanine in DNA does not constitute a barrier to transcription, but is converted into transcription-blocking damage by OGG1. Nucleic Acids Res..

[88] Allgayer J., Kitsera N., von der Lippen C., Epe B., Khobta A. (2013). Modulation of base excision repair of 8-oxoguanine by the nucleotide sequence. Nucleic Acids Res..

[89] Lühnsdorf B., Epe B., Khobta A. (2014). Excision of uracil from transcribed DNA negatively affects gene expression. J. Biol. Chem..

[90] Kitsera N., Allgayer J., Parsa E., Geier N., Rossa M., Carell T., Khobta A. (2017). Functional impacts of 5-hydroxymethylcytosine, 5-formylcytosine, and 5-carboxycytosine at a single hemi-modified CpG dinucleotide in a gene promoter. Nucleic Acids Res..

[91] Eckstein F. (2014). Phosphorothioates, essential components of therapeutic oligonucleotides. Nucleic Acid Ther..

[92] Matsumoto Y., Kim K., Bogenhagen D.F. (1994). Proliferating cell nuclear antigen-dependent abasic site repair in *Xenopus laevis* oocytes: An alternative pathway of base excision DNA repair. Mol. Cell. Biol..

[93] Matsumoto Y., Kim K., Hurwitz J., Gary R., Levin D.S., Tomkinson A.E., Park M.S. (1999). Reconstitution of proliferating cell nuclear antigen-dependent repair of apurinic/apyrimidinic sites with purified human proteins. J. Biol. Chem..

[94] Woodrick J., Gupta S., Camacho S., Parvathaneni S., Choudhury S., Cheema A., Bai Y., Khatkar P., Erkizan H.V., Sami F. (2017). A new sub-pathway of long-patch base excision repair involving 5′ gap formation. EMBO J..

[95] Schomacher L., Han D., Musheev M.U., Arab K., Kienhöfer S., von Seggern A., Niehrs C. (2016). Neil DNA glycosylases promote substrate turnover by Tdg during DNA demethylation. Nat. Struct. Mol. Biol..

[96] Rahimoff R., Kosmatchev O., Kirchner A., Pfaffeneder T., Spada F., Brantl V., Müller M., Carell T. (2017). 5-Formyl- and 5-carboxydeoxycytidines do not cause accumulation of harmful repair intermediates in stem cells. J. Am. Chem. Soc..

[97] Barbado C., Córdoba-Cañero D., Ariza R.R., Roldán-Arjona T. (2018). Nonenzymatic release of N7-methylguanine channels repair of abasic sites into an AP endonuclease-independent pathway in *Arabidopsis*. Proc. Natl. Acad. Sci. USA.

[98] Osman F., Bjørås M., Alseth I., Morland I., McCready S., Seeberg E., Tsaneva I. (2003). A new *Schizosaccharomyces pombe* base excision repair mutant, *nth1*, reveals overlapping pathways for repair of DNA base damage. Mol. Microbiol..

[99] Alseth I., Korvald H., Osman F., Seeberg E., Bjørås M. (2004). A general role of the DNA glycosylase Nth1 in the abasic sites cleavage step of base excision repair in *Schizosaccharomyces pombe*. Nucleic Acids Res..

[100] Sugimoto T., Igawa E., Tanihigashi H., Matsubara M., Ide H., Ikeda S. (2005). Roles of base excision repair enzymes Nth1p and Apn2p from *Schizosaccharomyces pombe* in processing alkylation and oxidative DNA damage. DNA Repair.

[101] Nilsen L., Forstrøm R.J., Bjørås M., Alseth I. (2012). AP endonuclease independent repair of abasic sites in *Schizosaccharomyces pombe*. Nucleic Acids Res..

[102] Grin I.R., Mechetin G.V., Kasymov R.D., Diatlova E.A., Yudkina A.V., Shchelkunov S.N., Gileva I.P., Denisova A.A., Stepanov G.A., Chilov G.G. (2021). A new class of uracil–DNA glycosylase inhibitors active against human and vaccinia virus enzyme. Molecules.

[103] Ran F.A., Hsu P.D., Wright J., Agarwala V., Scott D.A., Zhang F. (2013). Genome engineering using the CRISPR-Cas9 system. Nat. Protoc..

[104] Brinkman E.K., Chen T., Amendola M., van Steensel B. (2014). Easy quantitative assessment of genome editing by sequence trace decomposition. Nucleic Acids Res..

