# Supplementary material for: Back-Up Base Excision DNA Repair in Human Cells Deficient in the Major AP Endonuclease, APE1
Source: Int J Mol Sci. 2023 Dec 20;25(1):64. doi: 10.3390/ijms25010064 (PMC10778768; doi:10.3390/ijms25010064)
Supplement: Supplementary file 1 [file ijms-25-00064-s001.zip › ijms-2748035-supplementary.pdf]

Supplementary Figures S1–S3.

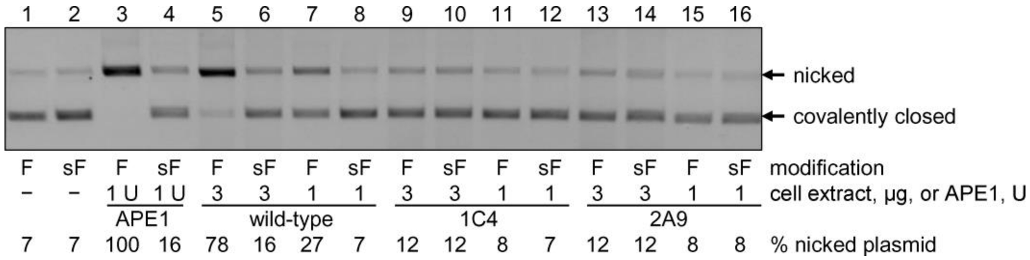

Supplementary Figure S1. Cleavage of F- and sF-containing plasmids by extracts of wild-type and *APEX1*^{KO} HEK293FT cells.

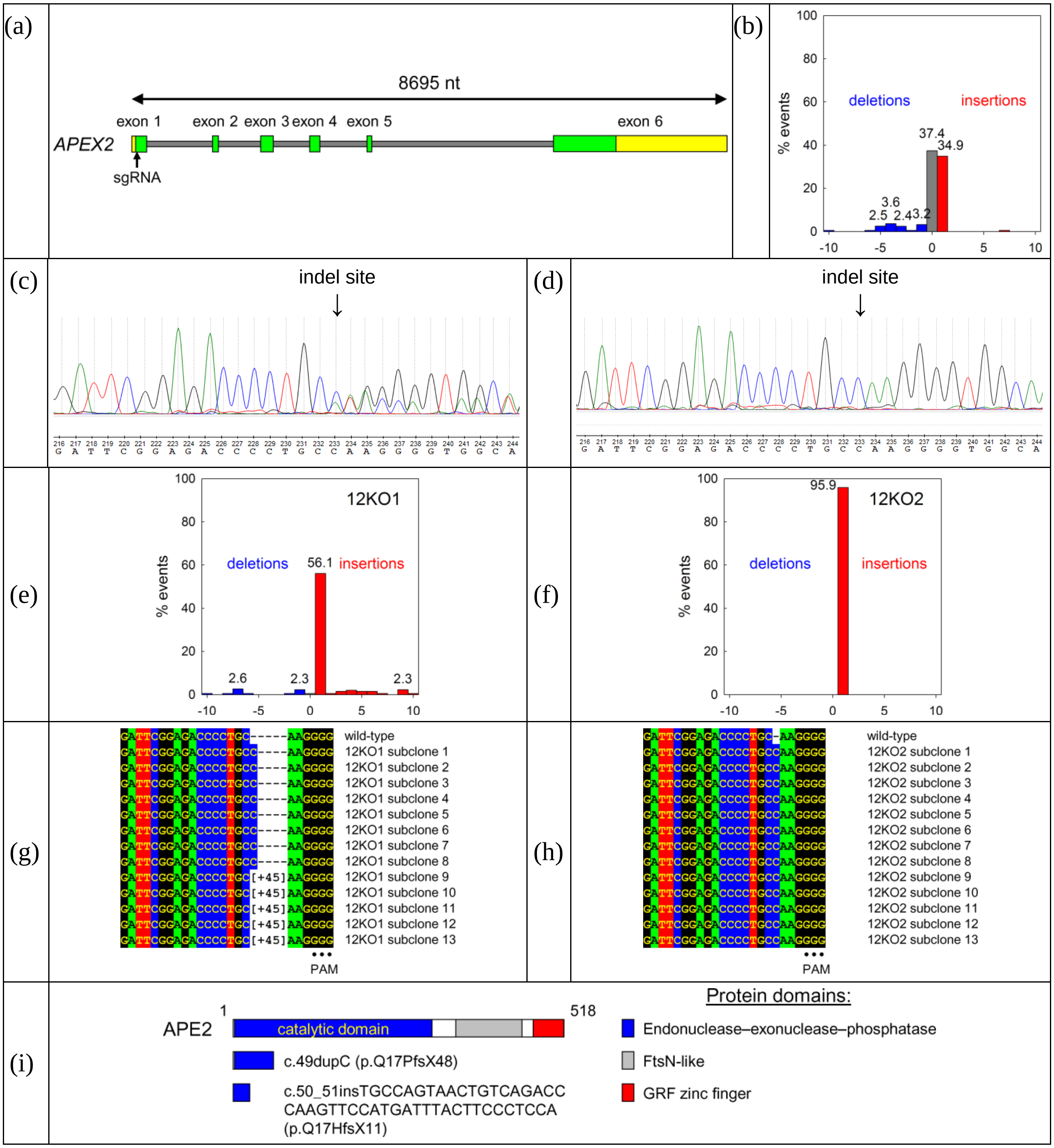

(a)
8695 nt
exon 1
exon 2
exon 3
exon 4
exon 5
exon 6
APEX2
sgRNA
(b)
% events
deletions
insertions
37.4
34.9
3.6
2.5
2.4
3.2
(c)
indel site
(d)
indel site
(e)
12KO1
% events
deletions
insertions
56.1
2.6
2.3
2.3
(f)
12KO2
% events
deletions
insertions
95.9
(g)
wild-type
12KO1 subclone 1
12KO1 subclone 2
12KO1 subclone 3
12KO1 subclone 4
12KO1 subclone 5
12KO1 subclone 6
12KO1 subclone 7
12KO1 subclone 8
12KO1 subclone 9
12KO1 subclone 10
12KO1 subclone 11
12KO1 subclone 12
12KO1 subclone 13
[+45]
PAM
(h)
wild-type
12KO2 subclone 1
12KO2 subclone 2
12KO2 subclone 3
12KO2 subclone 4
12KO2 subclone 5
12KO2 subclone 6
12KO2 subclone 7
12KO2 subclone 8
12KO2 subclone 9
12KO2 subclone 10
12KO2 subclone 11
12KO2 subclone 12
12KO2 subclone 13
PAM
(i)
APE2
1
518
catalytic domain
c.49dupC (p.Q17PfsX48)
c.50_51insTGCCAGTAACTGTCAGACC
CAAGTTCCATGATTTACTTCCCTCCA
(p.Q17HfsX11)
Protein domains:
Endonuclease–exonuclease–phosphatase
FtsN-like
GRF zinc finger

(j)

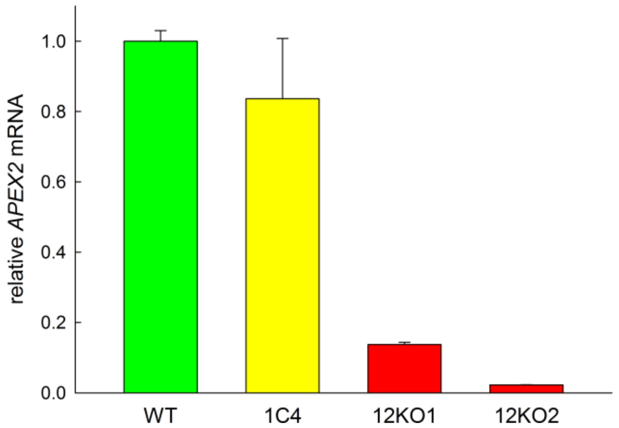

Supplementary Figure S2. Generation of *APEX1*^{KO} *APEX2*^{KO} cells. (a) Map of the human *APEX2* gene and location of the protospacer targeted by sgRNA. Green parts of the exons correspond to the protein-coding sequence. (b) TIDE analysis of the distribution of mutation events in the pool of 1C4 cells after transfection with pX458 expressing the *APEX2*-targeting sgRNA. The blue bars represent deletions, the red bars, insertions, the grey bar, sequences with no mutations. The X axis shows the distance from the target cleavage site in nucleotides. (c, d) Representative capillary sequencing chromatograms (ABI 3130xl instrument) of the PCR-amplified part of the *APEX2* gene from (c) 12KO1 and (d) 12KO2 monoclones. (e, f) TIDE analysis of the distribution of mutation events in (e) 12KO1 and (f) 12KO2 monoclones. (g, h) Sequences of the protospacer region in subcloned fragments from (g) 12KO1 and (h) 12KO2 monoclones. PAM, protospacer-adjacent motif. (i) Truncated APE1 proteins arising from the identified insertions at nucleotide 49. (j) Relative *APEX2* mRNA amount in *APEX1*^{KO} *APEX2*^{KO} cells. Mean and s.d. is shown (n = 2–4).

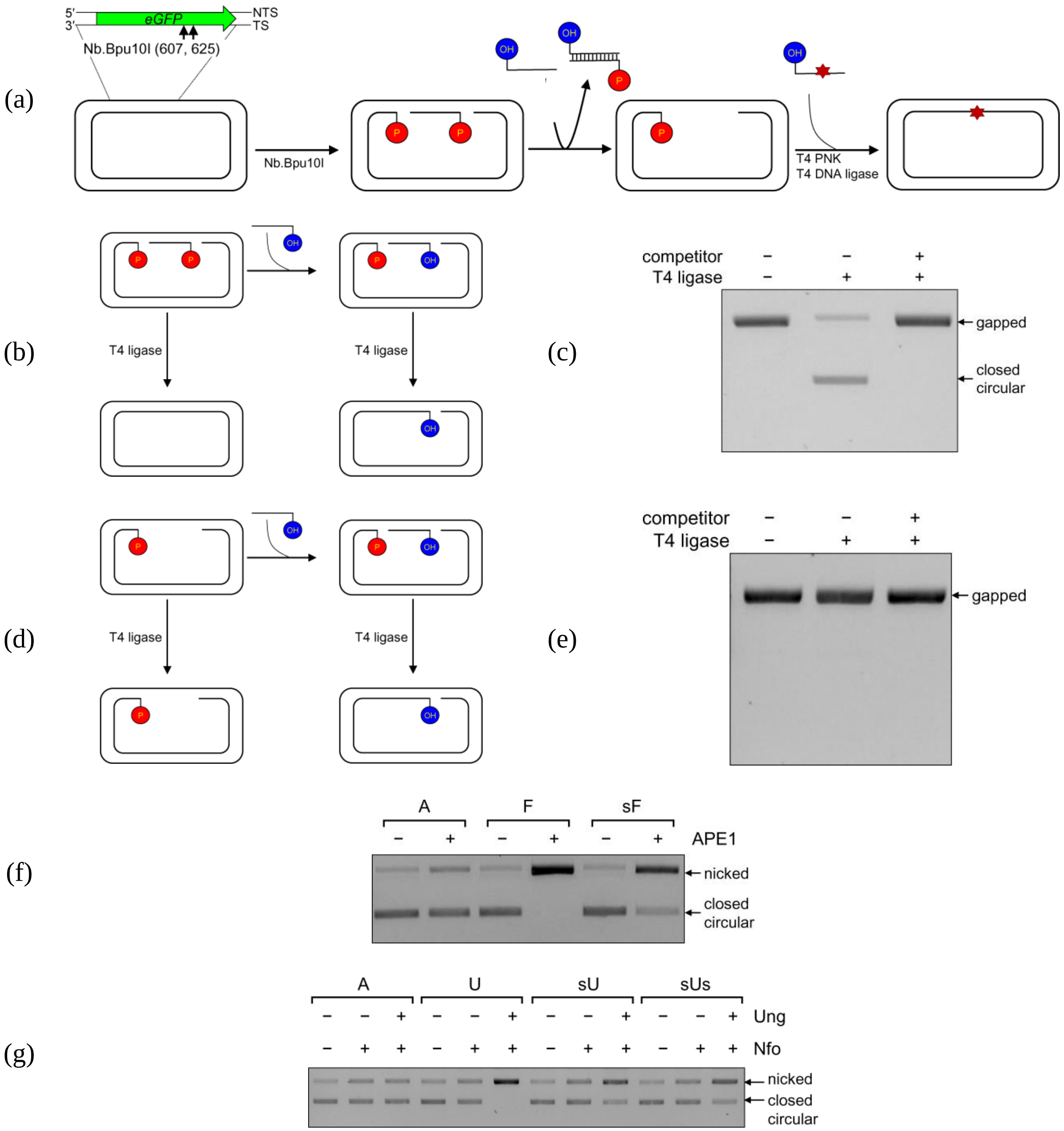

Supplementary Figure S3. Construction and quality control of the damage-containing plasmids. (a) General scheme of plasmid construction. Here and below, when relevant, phosphate and hydroxyl termini are marked with red and blue circles, respectively. Star indicates the damaged nucleotide. (b) Scheme of analytical ligation to confirm double nicking after cleavage with Nb.Bpu10I. (c) Representative gel image of analytical ligation of pZAJ_Q205* plasmid after cleavage with Nb.Bpu10I. (d) Scheme of analytical ligation to confirm gap formation after cleavage with Nb.Bpu10I and competitor nucleotide annealing. (e) Representative gel image of analytical ligation of pZAJ_Q205* plasmid after cleavage with Nb.Bpu10I and competitor nucleotide annealing. (f) Cleavage of the ligated damage-containing plasmid by APE1. (g) Cleavage of the ligated damage-containing plasmid by Ung and Nfo. The nature of the lesion in (f) and (g) is indicated over the gel image.
